# Treatment of Six Year Molars

**Published:** 1864-08

**Authors:** W. H. Atkinson


### TREATMENT OF SIX YEAR MOLARS.

Read before the Brooklyn Dental Association, April 29th, 1864.

BY W. H. ATKINSON.

When these teeth are largely decayed, they require large fillings to secure them from destruction, and hence the conducting power of the material used to fill them becomes an important consideration to him who is bound to save them ALIVE!

There are two objections to the exclusive use of gold for this purpose, besides want of the requisite manipulative skill, viz.:—The high degree of the power of conducting thermal and electric currents possessed by this otherwise desirable metal, and the high price it commands.

The former may be obviated by a judicious use of non-conducting substances between the main body of the filling and the point of proximity to the pulp chamber. One which I esteem as least objectional and most secure for this purpose is gold plate of the proper size, slightly concave on the side toward the pulp, to admit of a sufficient thickness of Hill's Stopping to intervene so as to completely cut off thermal and electric currents. I have seen good results from osteo-plastic non-conductors over which gold was securely packed.

The other objection is one not so easily overcome, as it involves a tender point to patient and practitioner, and involves a sacrifice of old notions to properly estimate the value of these teeth, on the part of patients, parents and guardians, or a compromise of the time, patience and skill, or a very large exercise of continued generosity on the part of the practitioner.

I therefore shall pass this point without imperious injunction, merely suggesting the *best way* as laying in the direction of saving *all* teeth by *all* the means necessary to that paramount end to be attained, trusting a generous providence for the consequences to our heavily taxed exchequer. He whose highest object is the money never renders the highest sort of service in any professional performance.

What, think ye, would the physical proportions of our children be if the principal stimulus to activity were the desire to be well-proportioned and fully developed, and they felt no spontaneous prompting impelling them to increase by loving to expand the energies of being?

In like manner we shall be dwarfed in the exact ratio that we exercise calculations of how soon we may make a stipulated amount of cash and how nicely we may balance our duties to our receipts, to the exclusion of feeling that our profession is a mission committed to us for the good of those we serve, the glory of God and a continual joy to our hearts that we have been accounted worthy to be honorably, because usefully, "recorded" among its martyrs, priests and performers.

Let no one who is not able to put forth his highest effort in the studies and practice of saving "six year molars" even entertain for a moment the proposition of entering our calling! For if it be not the chief purpose of his life to become first in preparation and competency of skill, he can not be happy in the exercise of that which he feels to be a sacrifice of his powers, which he would rather put forth in a direction more pleasant to himself.

All half-way operations grow out of half preparation or second choice in taking a part among us, both of which are sins to be deeply repented of, if we are to hope to grow out of their wretched consequences.

Why is it that so many of our body dread to encounter diseased "six year molars?" Simply because we have not inaugurated a habit of self-abnegation in a devotion to the highest good of those so unfortunate as to have contracted this deprecated malady; and because we have not trained ourselves to make it our highest pleasure and pastime to enter heartily into the demonstration of diagnosis, prognosis, and redemption of the aberrant organs to the joy of their possessor, and to the great increase of our own consequence and reputation among those doting parents hitherto utterly unable to obtain such a service for their dear charge!

Extraction and mere make-shift filling—any thing to get rid of the troublesome and difficult case—has too long held sway among us. The time has come when we must do our works of redemption faithfully, thoroughly and effectually, or we shall be forced to give place to those who will thus do it. So we may as well settle it promptly for ourselves whether we are to remain dead weights upon progressive dentistry, come up to the requirements made upon us, or be driven in disgrace from the pale of a profession too noble longer to tolerate such blundering in those claiming to be its exponents and practitioners. If I were asked what he is to do who desires to save "six year molars," of all the children of his charge, I would say I have briefly indicated the only state of mind and preparation by which it is possible to compass the end so devoutly to be wished; and that is, first, to have the heart so imbued with the high purpose that nothing short of full knowledge will satisfy him, and he is in a fair way to grow in ability of understanding, managing and preventing or remedying the diseases to which the "six year molar" is liable.

Do you ask why so urgent in preparation to save these teeth? Because these are and have been the opprobrium of dental practice. Also, that when the difficulties in the way of wholesale salvation of the "six year molar" shall have been removed, we have a clear field and plain path to the complete conservation or redemption of every other tooth of importance in the human mouth.

